# The relation between cochlear implant programming levels and speech perception performance in post-lingually deafened adults: a data-driven approach

**DOI:** 10.1007/s00405-023-08195-3

**Published:** 2023-09-04

**Authors:** Enrico Migliorini, Bastiaan van Dijk, Birgit Philips, Emmanuel Mylanus, Wendy Huinck

**Affiliations:** 1 Cochlear Technology Centre Belgium, Mechelen, Belgium; 2 Department of Otorhinolaryngology, Donders Institute for Brain, Cognition and Behaviour, Radboud University Medical Center Nijmegen, Nijmegen, The Netherlands; 3 Cochlear Benelux NV, Mechelen, Belgium

**Keywords:** Deafness, Cochlear implants, Retrospective, Speech perception, Prosthetics fitting

## Abstract

**Purpose:**

Programming a cochlear implant (fitting) is an essential part of a user’s post-implantation journey, defining how sound will be translated into electrical stimulation and aiming to provide optimal speech perception outcomes. Currently, there are no established, evidence-based guidelines for fitting cochlear implant users, leading to a high degree of variability in fitting practices, users’ parameters, and probably outcomes. In this study a data-driven approach is used to retrospectively investigate the relation between cochlear implant fitting parameters and speech perception outcomes in post-lingually deafened adults.

**Methods:**

298 data points corresponding to fitting parameters and speech audiometry test results for the same number of adult, post-lingually deafened, experienced CI users were analyzed. Correlation analysis was performed, after which parameters from the top-scoring and bottom-scoring tertiles were compared via the Mann–Whitney–Wilcoxon *u* test.

**Results:**

Weak correlations between dynamic range and speech audiometry outcomes were identified, having *p* values lower than (albeit close to) 0.05. A significant (*p* < 0.05) difference in electrical dynamic range (the difference between the minimum and maximum amount of current which may be delivered by each electrode) was found, with top-scoring subjects having on average a wider dynamic range.

**Conclusion:**

The association between dynamic range and speech perception outcomes shown in this retrospective study highlights the need for deeper investigation into evidence-driven fitting. It might be a first step in the direction of evidence-based fitting, minimizing variability in outcomes for cochlear implant users and helping mitigate the issue of unexplained low performance.

## Introduction

Outcome variability in adult cochlear implant (CI) users is a widespread and well-known problem [1–3], meaning that some CI users reach lower speech perception levels than expected, without a discernible reason[4–6].

Many research groups have focused on investigating the relationship between speech recognition, either in quiet or in noise, and factors, such as cause of deafness [7, 8], duration of deafness [3, 9, 10] and age at implantation [9, 11, 12]. Meaningful correlation was found in several cases [7–12], but these factors are of limited use for the purpose of addressing performance variability, as they cannot be intervened upon. One factor which, conversely, can be intervened upon, is the programming of sound processors. This is done by adjusting the so-called MAP, which is a structure made up of a large number of variables governing how sounds are translated into electrical pulses. Typical MAP parameters are, for instance, the pulse rate (number of impulses per second), the frequency allocation table (allocating a band of frequencies to each electrode) or the stimulation mode (e.g., monopolar or bipolar). In this study, the focus was placed solely on the upper and lower stimulation levels that are set per electrode. These two values determine the lower and upper bounds for how much electrical charge the implant will deliver to the cochlea at any given time. In Cochlear^®^ Nucleus^®^ devices, these values are referred to as T-levels and C-levels (from “threshold” and “comfort”, respectively).

Proper setting of T- and C-levels has been proven to be of fundamental importance for a user’s performance [13, 14]. A too small dynamic range (DR), which can be caused by setting T-levels too high or C-levels too low, will cause compression, leading to reduced speech recognition [15, 16]. Setting T-levels too low will lead to soft sounds being inaudible, whereas high C-levels will cause discomfort when exposed to loud sounds [14]. For the comfort of the user, it is also important to ensure that the perceived loudness is even across the various electrodes. Finding the correct values for these levels, or “fitting” as it is often called, is one of the most important components of a CI user’s journey after implantation, requiring experienced audiologists to perform gradual adjustments for potentially many years [4, 13]. With current clinical practices, there is no simple, programmatical way of fitting the MAP of a CI user.

Measurements such as the electrically evoked compound action potential (eCAP) and the electrically evoked stapedius reflex threshold (eSRT) can assist a clinician in fitting [17–20]; however, they still do suffer some drawbacks. ESRT measurement can be extremely loud and uncomfortable, not every clinic has a tympanometer and executing the eSRT measurement is not easy. Finally, eSRT cannot be used to predict T-levels. ECAP measurement can be useful to predict map shape but can also be loud and their relation to absolute T or C levels is weak.

All this leads to a large amount of variability in clinical fitting practices between different centers [13], with different clinics adopting different measures, different timelines, and different targets. For these reasons, fitting has sometimes been informally described as “more an art than a science”. Due to the aforementioned variability in practices and the lack of universally accepted fitting guidelines (i.e., standard of care), fitting parameters show a high degree of variability [21] across different centers and clinicians. Therefore, the question arises: what is the effect of this variability on performance?

Overall, in current literature on how fitting parameters relate to speech performance several topics seemed to be unexplored. First and foremost, there appears to be a shortage of observational studies investigating the relationship between performance and parameters of cochlear implants without conducting interventions. Second, within the interventional studies that have been performed, there seems to be a lack of research tailoring the interventions to the individual users, with most instead opting to apply the same intervention to every test subject. Given the high number of factors which contribute to speech perception, such an approach runs the risk of hiding confounding factors and diluting effects which may have a significant impact in subgroups or single individuals. Finally, no previous study focused specifically on low performers and how their MAPs differ from high performers’. As they make up the population who stands to gain the most from novel approaches to fitting, it could be worthwhile to run clinical studies keeping in consideration the specific issues that low performers may be facing. It should also be noted how high and low performance may be subjective, based on the expectations of the user, the pre-implantation condition, the population of users in the clinic, etc. For the purpose of the current study, it was decided to consider low performers the lower-scoring 33% of CI recipients in the context of a single clinic (as detailed in the “Methods” section).

As a result, the present study is conducted to investigate how T- and C-levels might be related to speech recognition outcomes by exploratory analysis of clinical data from adult post-lingually deafened cochlear CI users from Radboud university medical center. The aim is to use this knowledge to contribute to the development of more universal fitting guidelines.

## Methods

An anonymous data set of 298 data points was collated from clinical data collected at Radboud university medical center between the years 2006 and 2019. The data included the speech perception scores of 298 adult CI users, measured at 12 month post-implantation and the corresponding fitting MAP and related fitting parameters. The database was filtered to only include adults with bilateral, post-lingual deafness who were at least 18 years at implantation, implanted with a Cochlear^®^ Nucleus^®^ device, and using a modern sound processor (Freedom^®^, Nucleus^®^ 5 CP800, Nucleus^®^ 6 CP910 or Kanso^®^). Each data point includes as a dependent variable the results of the Dutch NVA Speech Audiometry test [22], i.e., a CVC (Consonant–Vowel–Consonant) test in the CI alone condition at an intensity of 65 dB SPL (Free field). Each data point also includes, as an independent variable, complete information about the MAP that was in use at the time of testing. This MAP information includes every non-identifying parameter which is saved in CustomSound^®^ (the Cochlear^®^ software used for programming speech processors): e.g., speech coding strategy, number of maxima, T- and C-levels, electrode impedances, NRT measurements when present, Frequency Allocation table, pulse rate, and pulsewidth.

The key parameters of this study were T- and C-levels; these levels determine the upper and lower bound for the amount of current which may be delivered to the electrode at any given time. That is, sounds falling between two thresholds called T (threshold) and C (comfort), both measured in dB SPL, will be mapped to Current Levels (CLs, the unit of measure for current delivered in the CustomSound fitting software) falling between the T- and C-levels for each respective electrode, according to a monotonic function called “loudness growth function”. Sounds softer than the T-SPL will not trigger a current being delivered to the electrode and sounds louder than the C-SPL will be compressed to prevent discomfort [23].

**Fig. 1:**
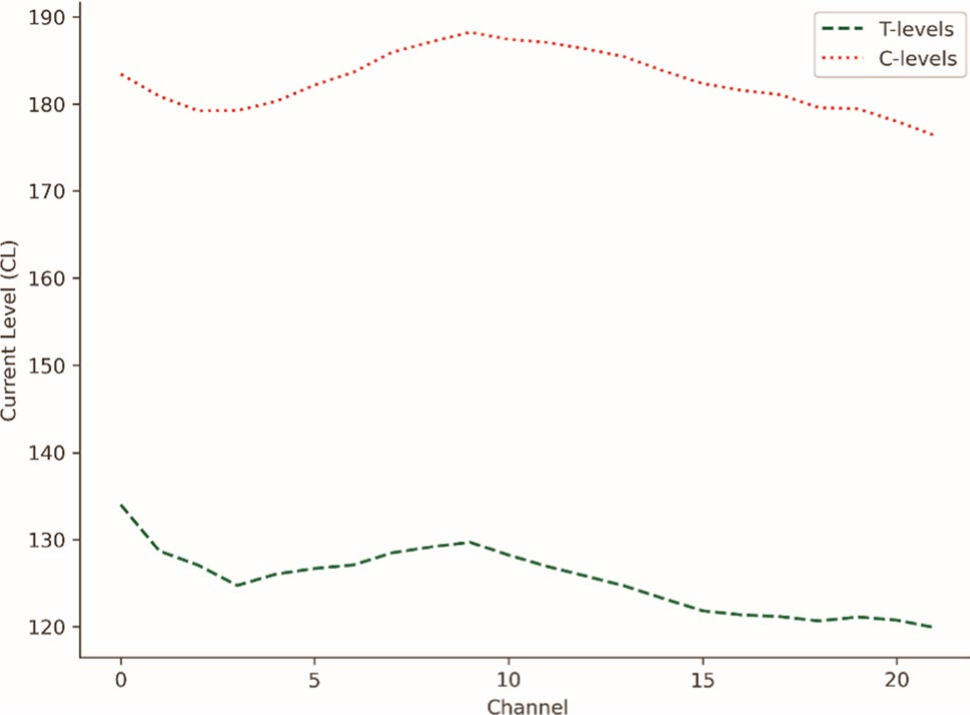
Average MAP for the population of this study, obtained by averaging T-levels and C-levels for all subjects. T-levels are shown in green, C-levels in red. The *X* coordinate goes from 22 to 1, because in the Nucleus﻿® systems, higher number channels code for lower frequencies. On the *Y* axis are current levels (CLs)

For MAPs where the pulsewidth was different from the standard 25 μs, T- and C-levels were scaled, so that values in CLs would be directly proportional to the amount of current delivered. The average T- and C-levels are shown in Fig. 1.

The next step was to reduce the dimensionality of the parameters. The exploration considered T- and C-levels as independent variables, and the speech audiometry outcome as the dependent one. As this results in each data point being 44-dimensional, it was decided to apply a dimensionality reduction algorithm to make data analysis more practical, specifically principal component analysis (PCA). As each PCA component is a linear combination of the 44 T- and C-levels, this technique serves a similar purpose as the analysis of aggregate values such as average levels or DR, the difference being that, instead of artificially defining the aggregate measure, the PCA algorithm provides them according to the amount of variance explained by each component. To properly deal with missing values corresponding to deactivated electrodes (in total, 182 out of 6556 electrodes were found to be deactivated, an average of 0.61 per subject), the variant algorithm of Probabilistic PCA [24] was used. This algorithm uses probabilistic estimation to impute missing data.

The number of components was selected based on the commonly used criterion of choosing as many components as needed to explain 95% of the data variance.

**Fig. 2:**
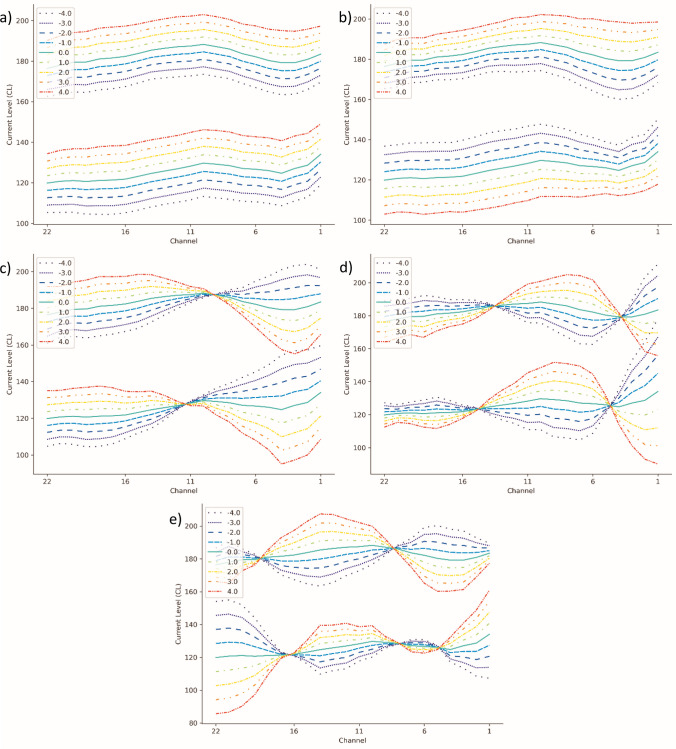
MAPs reconstructed from different PCA components. The *X* coordinate goes from 22 to 1 to mirror the display most commonly used by audiological software (i.e., low frequencies on the left). From **a**–**e**, components 1, 2, 3, 4, 5 are shown. Each line corresponds to a different value of the parameter a from which the MAPs are reconstructed, from − 4 to 4

Five components were sufficient to explain 95.03% of the original variance, so each 44-dimensional data point was converted into a 5-dimensional one, each component being a linear combination of the 44 original elements. As converting a data point into PCA components is a linear operation, it is also possible to invert it. This opens up the possibility of exploring what each component represents, i.e., how a change in T- and C-levels is expressed in terms of PCA components. To elaborate, the inverse PCA transformation of [0, 0, 0, 0, 0] results in a reconstructed MAP set to average values of T- and C-levels. The inverse transformation of [*a*, 0, 0, 0, 0] will show a hypothetical MAP which deviates from average in a way that translates to a change of *a* in the first PCA component. This allows to represent the five components’ effects on MAPs, as shown in Fig. 2, where hypothetical MAPs are reconstructed for levels of *a* ranging from − 4 to 4 for all five components separately.

The components appear to mirror MAP features. That is, a MAP with a high value for the first component can be expected to have higher average values for T- and C-levels than one with a lower first component. Similarly, the second component appears to be related to DR, the third one to the curve’s tilt, the fourth to its curvature (that is, the second-order derivative) and the fifth to its presence of double curvatures (third-order derivative).

Because of these similarities, it was decided to also define aggregate measures to be included in the analysis as independent variables; these measures are average T-level, average C-level, average DR, tilt (defined as first-order derivative of the values) of T-levels and tilt of C-levels. The rationale for including analysis of these human-defined measures was that guidelines based on easily understood aggregate parameters such as average levels should be easier to apply in clinical practice than using PCA components. Aggregate measures based on the fourth and fifth components were not investigated as it was unclear how to represent them mathematically.

After outliers were removed according to the interquartile rule and normality was checked with the Shapiro–Wilk test [25], the first step in analysis was to look for correlation between the MAP components and the subjects’ Phonemes Score. Both Pearson’s and Spearman’s coefficients were calculated, to account for both linear and non-linear correlation.

Subsequently two subgroups of data points were selected according to speech recognition, the first group consisting of the top 33% of tests based on the phonemes recognition score and the second one consisting of the bottom 33%. The thresholds were calculated to be at 85% phonemes recognition for the top tertile, and 71% for the bottom one. The rationale behind the use of tertiles was the desire to have a buffer between the top and bottom scores, so that data points close to the median value would not dilute the effects of the independent variables on higher and lower scores. After this subgrouping, the two-sided Mann–Whitney–Wilcoxon’s *u* test was used to look for significant differences in the distribution of PCA components values between the two groups. This was done to investigate whether some components had different distributions between the MAPs used in tests where the highest and lowest scores had been achieved. The choice of a non-parametric test was made after the assumption of normality had been found to be violated.

All the statistical analysis was done via Python, using the SciPy library. All of the pictures were produced via the Python libraries Matplotlib and Seaborn.

The top tertile included 100 data points, the bottom tertile included 94.

## Results

All the *p* values have been adjusted via Bonferroni–Holm correction [26, 27] to reduce the chance of potential false positives. This correction takes into account 30 different statistical tests. All values which, after correction, became higher than 1 have been adjusted to 1 as probabilities above 1 make no mathematical sense.

### Tests of normality

The Shapiro–Wilk test of normality returned values for the five PCA parameters, respectively, of 0.765, 0.003, 0.255, 0.060 and 0.694. As the second parameter turned out to deviate significantly from the normal distribution, non-parametric tests were chosen. For the aggregate measures of average T-level, average C-level, DR, T-level tilt and C-level tilt, the results were, respectively, 0.655, 0.445, 0.0004, 0.043, and 0.769. Again, the assumption of normality was violated, leading to the use of non-parametric tests.

### Correlation coefficients between the PCA components and phonemes recognition score

Table 1 shows the correlation coefficients and *p* values between the five PCA components and phonemes recognition score. No components show a significant correlation with speech performance score, although component 2’s *p* values could be considered borderline (*p* ≤ 0.1).

**Table 1:** Pearson’s and Spearman’s correlation coefficients (and *p* values) between the PCA component and phonemes recognition score for the PCA components

Component	Pearson’s coefficient	Pearson’s *p* value	Spearman’s coefficient	Spearman’s *p* value
Component 1	− 0.0158	1.0000	0.0188	1.0000
Component 2	0.1796	0.0546	0.1684	0.0975
Component 3	0.0762	1.0000	0.0799	1.0000
Component 4	0.1460	0.3432	0.0953	1.0000
Component 5	0.0668	1.0000	0.0398	1.0000

### Mann–Whitney–Wilcoxon tests for the PCA components

**Fig. 3:**
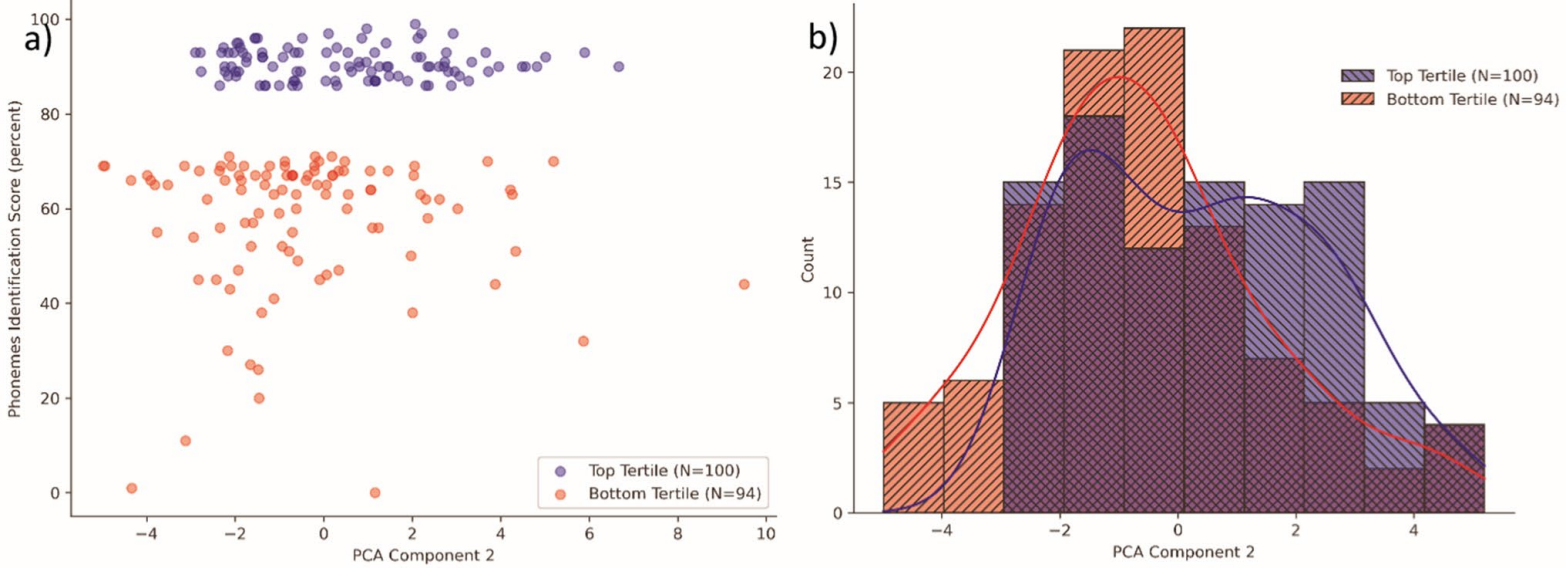
Differences in component 2 between the top and bottom tertiles. **a** Scatter plot of the two populations with PCA component 2 values on the *X* axis and phonemes recognition score on the *Y* axis. **b** Grouped data presented in two overlapping histograms with the kernel density estimation functions

Table 2 shows the results of the Mann–Whitney–Wilcoxon tests comparing the mean values of PCA components between the top scoring and bottom scoring tertiles. The only component for which the test had a *p* value lower than 0.05 was the second one, where a significant difference was observed between the top and bottom tertiles. Figure 3 shows the difference graphically.

**Table 2:** Results of the Mann–Whitney–Wilcoxon tests comparing PCA components between the top scoring and bottom scoring tertiles

Component	Mann–Whitney–Wilcoxon’s *u* statistic	Mann–Whitney–Wilcoxon’s *p* value
Component 1	4688.0	1.0000
Component 2	3581.0	0.0420
Component 3	4266.0	1.0000
Component 4	4107.0	1.0000
Component 5	4477.0	1.0000

The distribution of component 2 in the two populations seems to suggest that while a sizeable amount of data points in both tertiles fall in the [− 3, 0] interval, the [0, 3] interval contains more data points belonging to the top tertile.

### Correlation coefficients between the aggregate measures and phonemes recognition score

**Fig. 4:**
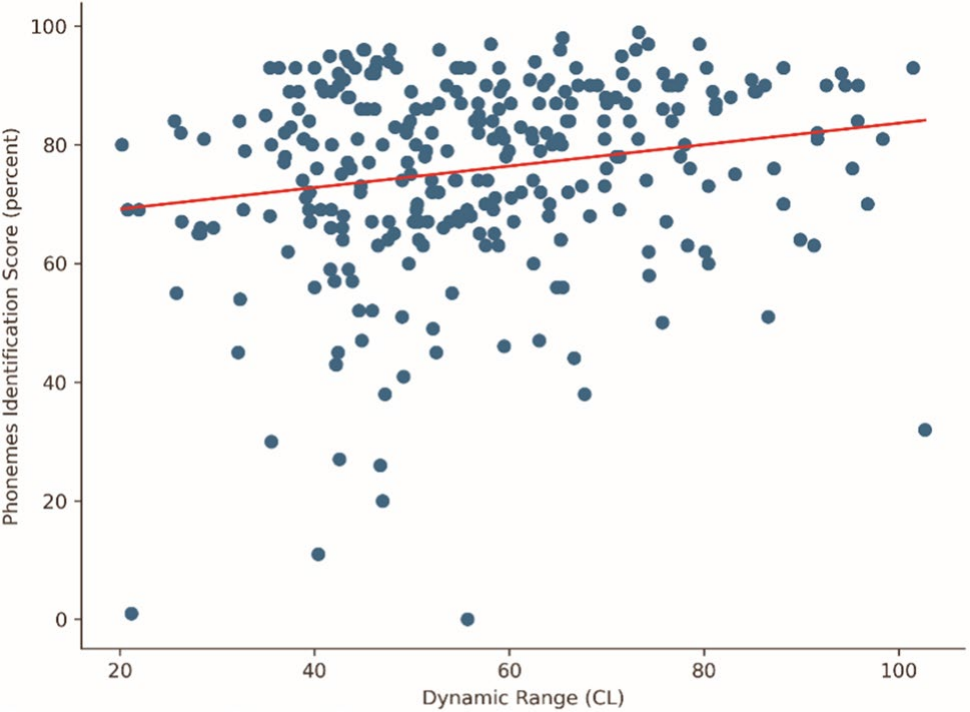
Scatter plot of DR against phonemes recognition score, including linear regression

A significant correlation was found between average DR and speech recognition outcomes. Table 3 shows the correlation coefficients for the aggregate measures. This correlation appears to be weak, as can also be seen in Fig. 4.

**Table 3:** Pearson’s and Spearman’s correlation coefficients and *p* values between the aggregate measures and phonemes recognition score

Measure	Pearson’s coefficient	Pearson’s *p* value	Spearman’s coefficient	Spearman’s *p* value
Average T-level	0.0881	1.0000	− 0.0738	1.0000
Average C-level	0.0441	1.0000	0.0740	1.0000
Average DR	0.1871	0.0377	0.1841	0.0420
Tilt of T-levels	0.1124	1.0000	0.0818	1.0000
Tilt of C-levels	0.0870	1.0000	0.0895	1.0000

### Mann–Whitney–Wilcoxon tests for the aggregate measures

**Fig. 5:**
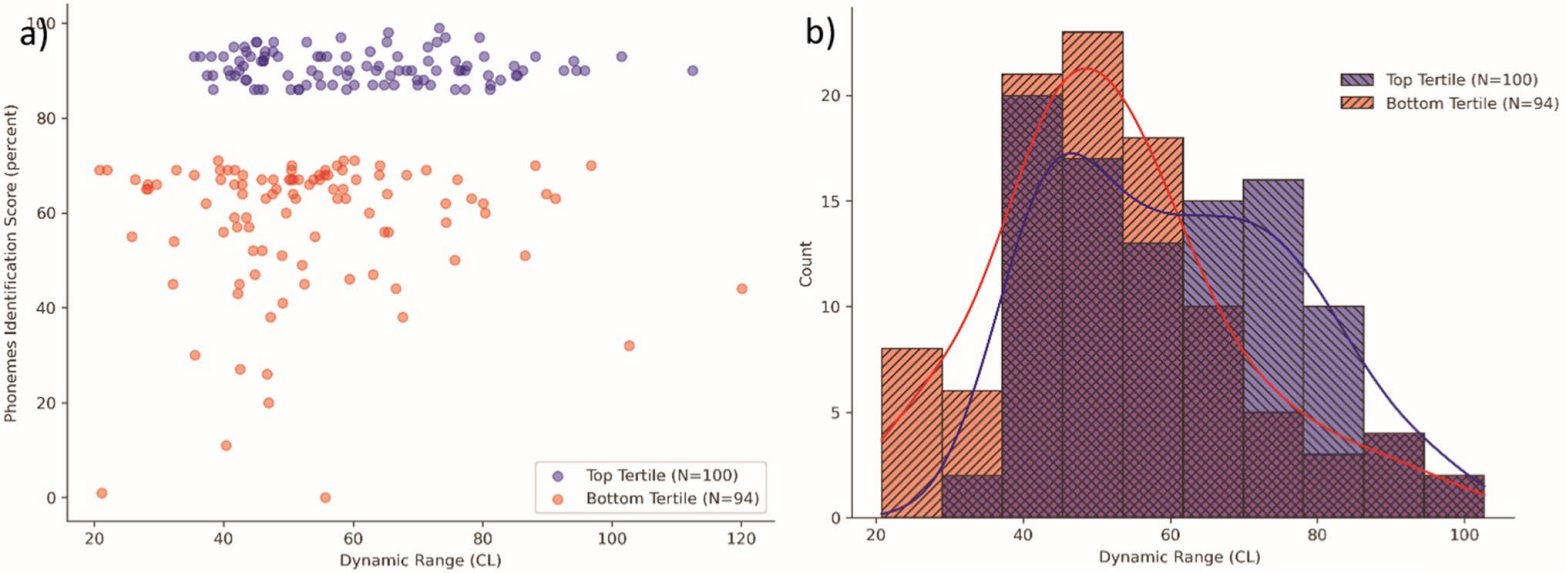
Differences in average DR between the top and bottom tertiles

Table 4 shows the results of the Mann–Whitney–Wilcoxon tests comparing the mean values of aggregate measures between the top scoring and bottom scoring tertiles. The only component for which the test indicated a significant difference in distribution between the top and bottom tertiles was the average DR, again mirroring the results obtained for PCA component 2. Figure 5 presents the distribution of DR both as a scatter plot and as histograms.

**Table 4:** Results of the Mann–Whitney–Wilcoxon tests comparing aggregate measures between the top scoring and bottom scoring tertiles

Measure	Mann–Whitney–Wilcoxon’s *u* statistic	Mann–Whitney–Wilcoxon’s *p* value
Average T-level	5323.5	1.0000
Average C-level	4249.0	1.0000
Average DR	3561.0	0.0180
Tilt of T-levels	4302.0	1.0000
Tilt of C-levels	3967.5	1.0000

The analysis of the average DR seems to confirm what appeared from the analysis of component 2: most of the bottom tertile falls approximately between 40 and 60 current levels of average DR. While many data points in the top tertile also fall in that interval, the interval between 60 and 80 sees many more data points from the top tertile than from the bottom, enough to cause a significant difference in averages. The average DR for the bottom tertile is 52.5 CL, for the top tertile it is 61 CL.

An initial assessment of the apparent subgroups in the top tertile was run, by selecting subjects above and below the median value of 59 CL to assess whether some factor may be associated with a wider DR in high performers. There were no significant differences in the distribution of implant type (Chi-squared statistic 0.078, *p* value 0.9616), processor (Chi-squared statistic 0.148, *p* value 0.9855), age at implantation (Mann–Whitney–Wilcoxon *u* 1346.5, *p* value 0.3968) or number of deactivated electrodes (Mann–Whitney–Wilcoxon *u* 1262.5, *p* value 0.7169).

### Statistical power analysis

The effect size *r* for the Mann-Whitney-Wilcoxon test was calculated to be 0.6200 for the second PCA component and 0.6041 for the DR. These values are commonly considered to correspond to a large effect size [28]. Given the sample sizes and the significance threshold being set at 0.05, this resulted in an achieved statistical power of 0.987 and 0.983, respectively. These values are commonly [29] considered to indicate a high power level.

## Discussion

This study investigated the relation between the most important fitting parameters (T- and C-levels) and speech recognition performance in adult post-lingually deafened Nucleus^®^ cochlear implant users. The main finding of this study is the significant association between DR and speech recognition performance in quiet in post-lingually deafened, Dutch-speaking, experienced CI users, with better-performing subjects showing a wider DR.

There are a number of possible explanations for these findings: perhaps they highlight how users who have good neural survival and can, therefore, adapt well to the CI might be able to handle a wider DR: in other words, having a healthy auditory system may result in high performance and in a wider DR. Another possibility is that users who have MAPs with a wider DR become accustomed to them and have improved amplitude modulation detection leading to higher levels of speech understanding. In other words, having a wider DR may lead one to be a better performer. Either or both of these possibilities may factor into overall speech understanding.

To explore the causality relationship, an intervention study could be designed with the goal of trying to widen the DR of CI users and checking whether this leads to significant improvements in performance. De Quilletes et al. [30] have presented preliminary results of an interventional trial which seems to support the hypothesis that the correlation found in this study could be used for a clinical intervention, but their work has not been published yet. Unlike the interventional studies which were mentioned in the introduction, studies of this sort would be informed by preliminary data analyses like the one presented in this paper, using learnings from the study population to give low performing users the MAP most suited to their condition, tailoring the intervention to the need of the population. It should be noted that this intervention cannot be expected to provide benefits to each subject, and this should be taken into consideration when designing it. In cases where a smaller DR is to be attributed to the physiological conditions of the auditory nerve, we cannot expect an increase in DR to yield better hearing quality to the CI user.

Furthermore, the distribution of DR in the top tertile seems to warrant further investigation into the characteristic of the population, as those with a DR between 60 and 80 appear to form a separate population from those with DR between 40 and 60. In the Results section, it was shown how implant, processor, age at implantation and number of deactivated electrodes did not differ in the two halves of the high performers group with the highest and lowest DR. This study could not examine clinical factors (e.g., aetiology, neural health or cognitive abilities) as the medical information of the recipients was inaccessible due to privacy regulations. There is also the possibility that ceiling effects were present in the high performers with a smaller DR: should their speech perception in quiet be satisfactory with a smaller DR, there would currently be no incentive for clinicians to increase the user’s DR. Future studies may want to investigate whether there are any clinical factors associated with wider DR and high performance, and whether clinical fitting guidelines might play a role in the creation of MAPs with smaller DRs.

A few papers have explored the relationship between T-levels, C-levels, and outcomes in adult CI users [16, 31–35]: in the two purely observational studies DR was shown to be associated with improved performance. Kim et al. [33] conducted a study on 40 adult subjects, 22 of which had a pre-lingual onset of their hearing loss and 18 of which had a post-lingual one. They found a weak but significant association between DR and a phonetically balanced word list test (*p* = 0.003, *r* = 0.462) as well as a consonant test (*p* = 0.005, *r* = 0.438). They formulated hypotheses on how reduced spectral peak recognition in users with a reduced DR might affect consonant recognition. Their overall conclusion was that further research was needed, as they did not consider their results to be conclusive. On the other hand, De Graaff et al. [34] investigated a group of 138 CI users with a post-lingual onset of deafness and found a consistently significant influence of DR on the performance in CVC word tests in quiet, but only in the population of subjects with late-onset deafness (where early onset included users implanted by the age of 7). These results were considered to be conclusive enough that the study brought forth recommendations for clinicians to aim for a DR between 40 and 60 current levels.

There can be several explanations why these different studies found mildly different results: for example, Kim’s study was carried out in the Republic of Korea, while De Graaff’s was run in the Netherlands. Differences in linguistics may perhaps account for some of the differences in the study results. In addition, the small size and heterogeneity of Kim’s study population may have impacted the study’s statistical power.

Several studies adopted a more interventional approach [16, 31, 32, 35]. These studies compared the subjects’ MAPs to ones that had undergone changes to test whether the changes in parameters had any influence on speech recognition. All the following studies selected experienced, postlingually deafened adults as their subjects.

Skinner et al. [31] tested raising minimum electrical thresholds by 2.04 dB in eight subjects, with the intervention resulting in improved speech recognition, achieving significant (*p* value < 0.05, with several instances of *p* < 0.01 and *p* < 0.001) results in CNC word score, CNC phoneme score and CUNY sentence score in babble at 50, 60, and 70 dB SPL presentation levels. Unfortunately, speech at lower levels was not measured. This is unfortunate particularly because low T-levels most likely affect lower levels. Busby and Arora [16] explored MAPs with different levels of expansion and compression of DR in 19 subjects on several words and sentences tests at different presentation levels. This study found no significant changes for expansion or compression of 30%, and a significant decrement (*p* < 0.001) in scores for expansion of 60% and 90%. Martins et al. [35] examined the performance of 30 post-lingually deafened adult CI users with MAPs having lower C-levels as well as ones having lower or higher T-levels. Lower C-levels resulted in worsened speech perception for both monosyllabic tests and sentence tests, both in quiet and in noise (*p* < 0.01); lower T-levels resulted in a significant worsening of scores for words in noise (*p* < 0.05) and higher T-levels in both reduced scores for sentences in noise (*p* < 0.05) and increased scores for words in quiet (*p* < 0.05). It is worth noting that all of the above-mentioned studies employed a broadband (i.e., overall) change in T- or C-levels which likely has little effect on between-band balance of loudness for small changes. This could explain the surprisingly large changes that were allowed without affecting the speech perception as was, for example, found by Busby et Arora [16].

Comparing the results of the present study with the state of the art, our results are similar to those found by De Graaff [34] and contrast those found by Kim [33]. As the choice to use PCA was made to have an unbiased overview of the tendencies in the datapoints before investigating the aggregate measures, the fact that these results mirror closely those found by De Graaff can, therefore, be seen as an independent validation of their investigation, as the present study converged to show similar tendencies. The contrast with Kim could be due, as postulated in the introduction, to differences in language between the subject population or to Kim’s study population, which was relatively small and heterogeneous as concerns onset of deafness.

Further research is needed to further investigate this relationship: investigating whether there is a causal relationship underlying the association of DR with speech understanding is of primary importance, as it may lead to innovative therapeutic interventions. The fact that both the present paper and De Graaff’s used data from the Netherlands can also be considered as a weakness: a larger, international multi-centre study may provide deeper insights on which MAP parameters are more relevant to native speakers of different languages. Finally, it is well-known that significant fitting differences exists between clinics [13] so results could in principle differ between clinics, i.e., clinics that are more cautious in setting C-level will have smaller DRs already on average so for them the effect could be stronger. Therefore, it is essential to repeat this kind of analysis over larger data sets, including different clinics and different clinicians. In addition, an investigation akin to the one described in this study should be performed for Speech in Noise tests, since differences in Dynamic Range might impact outcomes in noise, as suggested by a paper by Zirn et al. [36].

Present findings might provide useful data for audiologists during clinical fitting. Specifically, while it is often recommended to set a DR between 40 and 60 CL [34], it may be worthwhile to provide low performing CI users with MAPs which progressively increase the DR as this study suggests the possibility that some CI users may have MAPs with a smaller DR than what would be optimal for them.

There is an additional benefit in conducting further data analyses. The field of machine learning (ML) has flourished in the last two decades, bringing sweeping, radical innovations to multiple disciplines; among others, cochlear implant fitting has been impacted. Multiple papers have explored ML-based fitting techniques: genetic algorithms were tested in several studies [37–40], the use of data from past patients to generate MAPs for new ones was investigated [41] and the automated Fitting to Outcome eXperts (FOX) tool has shown considerable potential for suggesting beneficial changes in MAPs [42–45]. As the name implies, machine learning focuses on the creation of agents which are capable to learn, and to do so these systems require vast amounts of data points. Therefore, data analysis studies such as the one presented in this paper have the potential to inform the design of future ML system, anticipating the needs of future researchers.

The authors believe that the reduction of variability and a shift towards fitting practices that are deeply rooted into an evidence-based approach, whether by informing clinicians or by laying the groundwork for ML-based systems, will improve the speech perception outcomes for many CI users by helping their clinicians making better-informed choices stemming from a deeper understanding of how fitting influences outcomes. This will eventually lead to improved quality of life for CI users, as well as a more efficient clinical pathway.

## Conclusion

The association of DR with speech performance has been shown to be significant within our population. In the context of moving away from the current fragmented landscape of “fitting as an art”, where every clinic (or potentially every clinician) follows its own empirical guidelines, and towards an evidence-based approach, it is important that the relationship between fitting parameters and speech understanding is analyzed in the utmost detail.

Further research is required, due to the questions raised in the “Discussion” section. However, this early result could already be used to inform a clinical trial focused on evaluating the effectiveness of increasing DR in postlingually deaf CI users. Present study might be a first step in the direction of evidence-based fitting, minimizing variability in outcomes for cochlear implant users and helping mitigate the issue of unexplained low performance.

## Data Availability

Due to internal privacy rules of Radboud university medical center, data sharing is not allowed without explicit consent from all involved subjects, not even in anonymous form.

## Declarations

### Employment

Enrico Migliorini, Bas van Dijk and Birgit Philips are employed by Cochlear Limited.

## References

[1] Firszt JB (2004). Recognition of speech presented at soft to loud levels by adult cochlear implant recipients of three cochlear implant systems. Ear Hear.

[2] Gifford RH, Shallop JK, Peterson AM (2008). Speech recognition materials and ceiling effects: considerations for cochlear implant programs. Audiol Neurotol.

[3] Holden LK (2013). Factors affecting open-set word recognition in adults with cochlear implants. Ear Hear.

[4] Moberly AC, Bates C, Harris MS, Pisoni DB (2016). The enigma of poor performance by adults with cochlear implants. Otol Neurotol.

[5] Pisoni DB, Kronenberger WG, Harris MS, Moberly AC (2017). Three challenges for future research on cochlear implants. World J Otorhinolaryngol Head Neck Surg.

[6] Hoppe U, Hocke T, Hast A, Iro H (2021). Cochlear implantation in candidates with moderate-to-severe hearing loss and poor speech perception. Laryngoscope.

[7] Shearer AE (2017). Genetic variants in the peripheral auditory system significantly affect adult cochlear implant performance. Hear Res.

[8] Nishio SY (2022). Etiology of hearing loss affects auditory skill development and vocabulary development in pediatric cochlear implantation cases. Acta Otolaryngol.

[9] Beyea JA (2016). Cochlear implants in adults: effects of age and duration of deafness on speech recognition. Otol Neurotol.

[10] Goudey B (2021). A multicenter analysis of factors associated with hearing outcome for 2,735 adults with cochlear implants. Trends Hear.

[11] Mahmoud AF, Ruckenstein MJ (2014). Speech perception performance as a function of age at implantation among postlingually deaf adult cochlear implant recipients. Otol Neurotol.

[12] Heutink F (2021). Factors influencing speech perception in adults with a cochlear implant. Ear Hear.

[13] Vaerenberg B (2014). Cochlear implant programming: a global survey on the state of the art. Sci World J.

[14] Wolfe J, Schafer EC (2014) Programming cochlear implants. Plural Publishing [Online]. https://books.google.be/books?id=WiqDmAEACAAJ

[15] Loizou PC, Dorman M, Fitzke J (2000). The effect of reduced dynamic range on speech understanding: implications for patients with cochlear implants. Ear Hear.

[16] Busby PA, Arora K (2016). Effects of threshold adjustment on speech perception in nucleus cochlear implant recipients. Ear Hear.

[17] Smoorenburg GF, Willeboer C, Van Dijk JE (2002). Speech perception in nucleus CI24M cochlear implant users with processor settings based on electrically evoked compound action potential thresholds. Audiol Neuro-Otol.

[18] Kaplan-Neeman R (2004). Nrt-based versus behavioral-based map: a comparison of parameters and speech perception in young children. J Basic Clin Physiol Pharmacol.

[19] Potts LG, Skinner MW, Gotter BD, Strube MJ, Brenner CA (2007). Relation between neural response telemetry thresholds, T- and C-levels, and loudness judgments in 12 adult nucleus 24 cochlear implant recipients. Ear Hear.

[20] Holstad BA (2009). Relation of electrically evoked compound action potential thresholds to behavioral T- and C-levels in children with cochlear implants. Ear Hear.

[21] Wathour J, Govaerts PJ, Deggouj N (2021). Variability of fitting parameters across cochlear implant centres. Eur Arch Oto-Rhino-Laryngol.

[22] Bosman AJ (1989) Speech perception by the hearing impaired: (met een samenvatting in het Nederlands), Rijksuniversiteit Utrecht [Online]. http://books.google.nl/books?id=mUSONAAACAAJ

[23] Vaerenberg B, Govaerts PJ, Stainsby T, Nopp P, Gault A, Gnansia D (2014). A uniform graphical representation of intensity coding in current-generation cochlear implant systems. Ear Hear.

[24] Porta J, Verbeek J, Krose B, Porta J, Verbeek J, Kröse B (2005). Active appearance-based robot localization using stereo vision. Auton Robots.

[25] Shapiro SS, Wilk MB (1965). An analysis of variance test for normality (complete samples). Biometrika.

[26] Holm S (1979) A simple sequentially rejective multiple test procedure. Scand J Stat. http://www.jstor.org/stable/4615733. Accessed 7 Feb 2023

[27] Gaetano J (2018) Holm-Bonferroni sequential correction: an EXCEL Calculator—Ver. 1.3 [Online]. https://www.researchgate.net/publication/242331583_Holm-Bonferroni_Sequential_Correction_An_EXCEL_Calculator_-_Ver_12

[28] Fritz CO, Morris PE, Richler JJ (2012) Effect size estimates: current use, calculations, and interpretation. J Exp Psychol Gen 141(1):2–18. 10.1037/a0024338 10.1037/a0024338 21823805

[29] Jones SR, Carley S, Harrison M (2003). An introduction to power and sample size estimation. Emerg Med J.

[30] De Quillettes R, Kaandorp MW, Merkus P, Kramer SE, Smits C (2022) Improving adult Cochlear Implant users’ speech recognition performance in quiet and noise by using predefined fitting parameters. In: Presentation at the HEAL2022 conference

[31] Skinner MW, Holden LK, Holden TA, Demorest ME (1999). Comparison of two methods for selecting minimum stimulation levels used in programming the nucleus 22 cochlear implant. J Speech Lang Hear Res.

[32] Boyd PJ (2006). Effects of programming threshold and maplaw settings on acoustic thresholds and speech discrimination with the MED-EL COMBI 40+ cochlear implant. Ear Hear.

[33] Kim SY (2018). Electrical dynamic range is only weakly associated with auditory performance and speech recognition in long-term users of cochlear implants. Int J Pediatr Otorhinolaryngol.

[34] De Graaff F (2020). Relationship between speech recognition in quiet and noise and fitting parameters, impedances and ECAP thresholds in adult cochlear implant users. Ear Hear.

[35] Martins KVC, Goffi-Gomez MVS (2021). The influence of stimulation levels on auditory thresholds and speech recognition in adult cochlear implant users. Cochlear Implants Int.

[36] Zirn S, Polterauer D, Keller S, Hemmert W (2016). The effect of fluctuating maskers on speech understanding of high-performing cochlear implant users. Int J Audiol.

[37] Wakefield GH, Parkinson WS, Lineaweaver S, van den Honert C (2004) Recipient-directed design of speech processor MAPs. In: International congress series, vol 1273, no C, pp 178–182. 10.1016/j.ics.2004.09.003

[38] Wakefield GH, Van Den Honert C, Parkinson W, Lineaweaver S (2005). Genetic algorithms for adaptive psychophysical procedures: recipient-directed design of speech-processor MAPs. Ear Hear.

[39] Başkent D, Eiler CL, Edwards B (2007). Using genetic algorithms with subjective input from human subjects: implications for fitting hearing aids and cochlear implants. Ear Hear.

[40] Legrand P (2007). Interactive evolution for cochlear implants fitting. Genet Program Evolvable Mach.

[41] Torresen J, Iversen AH, Greisiger R (2017) Data from past patients used to streamline adjustment of levels for cochlear implant for new patients. In: 2016 IEEE symposium series on computational intelligence, SSCI 2016.10.1109/SSCI.2016.7850063

[42] Govaerts PJ, Vaerenberg B, De Ceulaer G, Daemers K, De Beukelaer C, Schauwers K (2010). Development of a software tool using deterministic logic for the optimization of cochlear implant processor programming. Otol Neurotol.

[43] Vaerenberg B, Govaerts PJ, De Ceulaer G, Daemers K, Schauwers K (2010). Experiences of the use of FOX, an intelligent agent, for programming cochlear implant sound processors in new users. Int J Audiol.

[44] Vaerenberg B, De Ceulaer G, Szlávik Z, Mancini P, Buechner A, Govaerts PJ (2014). Setting and reaching targets with computer-assisted cochlear implant fitting. Sci World J.

[45] Meeuws M, Pascoal D, Bermejo I, Artaso M, De Ceulaer G, Govaerts PJ (2017). Computer-assisted CI fitting: is the learning capacity of the intelligent agent FOX beneficial for speech understanding?. Cochlear Implants Int.

